# NexGenEx-Tom: a gene expression platform to investigate the functionalities of the tomato genome

**DOI:** 10.1186/s12870-014-0412-2

**Published:** 2015-02-13

**Authors:** Hamed Bostan, Maria Luisa Chiusano

**Affiliations:** Department of Agricultural Sciences, University of Naples “Federico II”, via Università 100, 80055 Portici, Italy

**Keywords:** Tomato expression database, NGS data analysis, Expression Atlas, Gene expression profiling, Gene expression comparison

## Abstract

**Background:**

Next Generation Sequencing technologies (NGS) unexpectedly pushed forward the capability of solving genome organization and of widely depicting gene expression. However, although the flourishing of tools to process the NGS data, versatile and user-friendly computational environments for integrative and comparative analyses of the results from the increasing amount of collections are still required.

The gene expression of tomato tissues has been widely investigated in the years, thanks to both EST sequencing and different microarray platforms. However, the resulting collections are heterogeneous in terms of experimental approaches, genotypes and conditions, making the data far from representing a gene expression atlas for the species. Therefore, the recent release of NGS transcriptome collections from several tissues and stages from physiological conditions for specific tomato genotypes provides a relevant resource to be appropriately exploited to address key questions on gene expression patterns, such as those related to fruit ripening and development in tomato. The organization of the results from the processed collections in web accessible environments, enriched with tools for their exploration, may represent a precious opportunity for the scientific research in tomato and a reference example for similar efforts.

**Description:**

Here we present the architecture and the facilities of *NexGenEx-*, a web based platform that offers processed NGS transcriptome collections and enables immediate analyses of the results. The platform allows gene expression investigations, profiling and comparisons, and exploits different resources.

Specifically, we present here the platform partition *NexGenEx-Tom*, dedicated to the organization of results from tomato NGS based transcriptomes.

**Conclusion:**

In the current version, *NexGenEx-Tom* includes processed and normalized NGS expression data from three collections covering several tissue/stages from different genotypes. Beyond providing a user-friendly interface, the platform was designed with the aim to easily be expanded to include other NGS based transcriptome collections. It can also integrate different genome releases, possibly from different cultivars or genotypes, but even from different species. The platform is proposed as an example effort in tomato, and is described as a profitable approach for the exploitation of these challenging and precious datasets.

## Background

Nowadays, the development of high throughput methodologies and advanced genomics are leveraging the genome-wide characterization of many different species [1]. Next Generation sequencing (NGS) technologies are strongly supporting this progress, being extensively exploited for both genome sequencing and gene expression investigations. These novel approaches play a key and unexpected role for their impressive capability to offer an in depth scanning of the transcriptome level of the cell functionality, providing a consistent contribution to the exploration of the genome expression, its control and related regulatory mechanisms. Moreover, NGS based transcriptome collections provide extended knowledge of gene structure and of expression of alternatively spliced gene models. Nonetheless, though these technologies are expanding, also because of their cost affordability [2], even to not specialized research centers, the large amount of data produced makes straightforward exploitations still a challenge for the scientific community.

Reference platforms such as Sequence Read Archive (*SRA*) [3] and Gene Expression Omnibus (*GEO*) [4] have been established to gather the NGS primary data collections, i.e. those directly provided by the sequencing centers in raw formats.

A significant number of tools and software have been released to pre-process (cleaning, trimming and quality checking [5,6]), process (*de novo* assembling [7,8], mapping to the genome sequence [9,10]), and analyze [11,12] the NGS data. Platforms including tools and pipelines provided with appropriate interfaces are flourishing to fulfill the need to support the transferring from raw data to accessible and meaningful biological information. Some interesting examples comes from the *Galaxy* project [13], which is a comprehensive open source toolbox meant to offer an interactive web based platform for running and tracing the manifold bioinformatics analyses useful for genomics, also permitting workflows on varied data types, NGS libraries included. Interestingly, the platform evolves thanks to a collaborative community of users and developers as a shared effort in an open source framework. *GenePattern 2.0* [14] provides access to a large number of tools not limited to RNA-seq analyses, also permitting the creation of multi-step pipelines, allowing the definition of appropriate workflows for reproducible analyses. *NGS-Trex* [15] is a web based platforms, exclusively dedicated to NGS data analysis, offering tools within a predefined workflow. *InSilico DB* [16] is a web based data storage and processing hub, free for basic usage and under payment for expanded analyses. It offers access to large pre-processed datasets from different species, but also methods for stand-alone genome wide analyses. The *CLC* bio integrated platform [17] offers a wide range of software and tools for biological data analyses. Processing toolboxes and computational power for RNA-seq data management in the form of online or stand-alone tools are available for free (Sequence viewer), or under payment, if requested in the form of workbenches.

In terms of visualizing NGS based results, the *Integrative Genomics Viewer (IGV)* [18] and *Gbrowse* [19] efficiently handle large heterogeneous datasets, while providing a smooth and intuitive user access to all levels of genome resolution. Both platforms support NGS data visualization in the form of short reads and coverage plots along genomic sequences.

Some other efforts specifically aim to provide immediate tools to investigate collections of processed data, for integration and data sharing. As an instant *RNASeqExpressionBrowser* [20] provides query interfaces by gene identifiers (ID), by keyword or by sequence, exploiting data results uploaded in a matrix-like format. There are also several, more specific reference sites, offering tools for the visualization of the gene expression results, mainly in the form of single dimension processed collections (plain tab delimited or csv formatted files including the genes and their expression levels as raw reads count or defined by specific normalization approaches) or in the form of reads mapped on the genome and accessible by a suitable genome browser interface. As an example, the *Ensembl* Genome Browser [21] provides NGS data for many differ species, in the form of short read tracks mapped onto the corresponding reference genome sequence and visually accessible from the associated genome browser interface, supporting scripting and APIs for in-depth comparative analysis and data mining [22].

In Plants, several resources are dedicated to species specific collections. The *TIGR* Rice Annotation project [23] includes data from RNA-seq collections offers tools for gene based investigations and for genome based views of their mapped distribution.

*SoyBase* [24], the USDA-ARS soybean genetic database, also includes RNA-seq expression data from a collection including different libraries, offering search, comparative and clustering tools for the analyses.

The Potato Genomic Resource (*Spud DB*) [25] also provides potato RNA-seq data in the form of raw short read sequence files and of FPKM normalized results as defined by the *Cufflinks* pipeline [26] (analogous to the RPKM normalization [27]) in Excel format, as well as visualization of expression tracks by library in a potato genome browser.

The Maize gene atlas [28] also provides a consistent RNA-seq collections of 18 tissues representing five organs. This dataset, together with microarray data from 60 unique spatially and temporally separated tissues from 11 maize organs, offers a comprehensive collection for understanding gene transcription during maize development.

An *RNA-seq expression atlas* [29] for common bean has also been recently released.

We here propose *NexGenEx-* as a bioinformatics platform designed to make pre-processed NGS based transcriptome collections available for suitable exploration of gene expression based on a reference genome sequence, when the corresponding gene annotation is available. In particular, as an example of application, we here describe the partition dedicated to tomato: *NexGenEx-Tom*.

Recently, the tomato genome sequencing Consortium released the preliminary version of the pseudomolecules of *S. lycopersicum*, the common tomato [30]. Tomato is the most extensively investigated *Solanaceae* species and it is a model system for fruit development. Defining its genome organization, its gene content and the related expression is relevant for addressing several open questions, such as the underlying mechanisms involved in fruit ripening and development, quality and yield. Moreover, these issues are also fundamental for comparative analyses with related genomes, such as those from wild relatives or from other *Solanaceae* species, like potato [31], pepper [32], Eggplant [33], or from other species with fleshy fruits, like grapevine. The 12 tomato pseudomolecules represent a first reference of the sequences of the tomato chromosomes, resulting in ~760 Mb (the expected total size is estimated to be ~900 Mb) [30]. Specifically, the Consortium sequenced the Heinz 1706 cultivar and also produced a collection of *Illumina* based transcriptome sequences from the same genotype. This collection includes two biological replicates from 10 different samples, from tissues and developmental stages in physiological conditions. The aim of the effort was to enrich the release of the genome sequence with a comprehensive overview of its expression, and to release preliminary results depicting the expression patterns associated to fruit settings and development. Moreover, RNA-seq data were also provided from different samples in physiological conditions of the putative closest wild relative *Solanum pimpinellifolium* [30]. Recently, similar data from different stages of fruit development from the genotype *Ailsa Craig* have been also provided [34].

The three NGS based collections are relevant if comparatively exploited since they offer insights on gene expression from several tissue/stages from different related genotypes, but also because they offer the opportunity to extensively investigate tissue specific gene expression patterns.

Several resources offer tomato gene expression data. The Tomato Functional Genomics Database (*TFGD*) [35] is a website specifically aimed to provide a representative resource for gene expression collections from tomato, including data from heterogeneous platforms (ESTs, microarrays, RNA-seq). Among different RNA-seq collections, the platform also organizes results from the three collections we described, offering RPKM normalized data per library when querying for a selected collection by gene ID or keyword.

The *Solanaceae* Genomic Network (*SGN*) website [36] is offering combined results from RNA-seq from unspecified collections in the form of short reads mapped onto the genome and accessible by a genome browser interface as coverage plots.

To our knowledge, the Tomato Genomic Resources Database (*TGRD*) [37] is the other tomato related resource also providing the RNA-seq based expression of tomato genes in selected tissues (leaf, root, flower and fruit) from the Heinz reference collection [30].

*NexGenEx-Tom* is dedicated to the organization of gene expression data from tomato, currently including the three publically available tomato collections from different genotypes, representing several tissues and stages from physiological conditions. As a novelty in the framework of tomato genomics, it offers different tools to explore and mine the processed and normalized data permitting straightforward, flexible analyses, abstracting results in different user-friendly views and offering the possibility to customize the investigations on single and/or multiple genes sets defined according to suitable queries.

In the current settings, *NexGenEx-Tom* offers a useful resource for tomato, because it permits to friendly explore the genome functionality and to straightforward investigate NGS based gene expression patterns in physiological conditions. This environment is designed to be easily expanded with new libraries and is ready for inclusions of different genomes, even from other species, representing a reference framework for similar approaches in other genomics contexts.

## Construction and content

### Platform architecture

**Figure 1:**
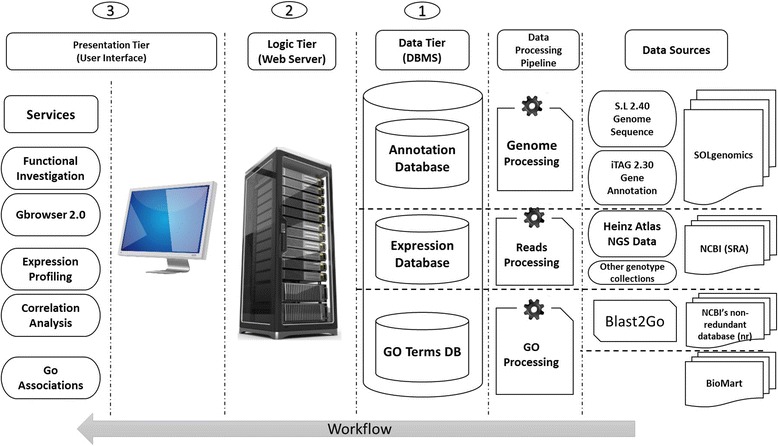
**Schema of the platform architecture and services offered.** The platform is implemented in a three-tier architecture schema: 1) Data Tier; 2) Logic Tier; 3) Presentation Tier. The schema also shows main data processing and services available and describes the actual reference sources.

We implemented *NexGenEx-* as a role based platform which enables the exploration of NGS based transcriptome collections. The platform was designed to provide enhanced tools for straightforward genome-wide gene expression analyses. As it is shown in Figure 1, the platform is implemented in a Three-tier Architecture schema: 1) Data Tier: Database Management System (DBMS) and Physical Storage Files; 2) Logic Tier: classes and modules deployed on the webserver, and 3) Presentation Tier: a Graphical User Interface (GUI) accessible to the end-users. Figure 1 also describes the main data processing pipelines necessary to define the data to be included in the platform and the resource collections from tomato here included.

The platform works as a web based application running on the .Net Framework 4.0, querying embedded databases, designed and organized in a relational model and implemented in *MySQL*, version 5.6.14 *InnoDB* engine [38]. All key fields and query dependent tuples were indexed using the *BTree* indexing algorithm [39]. A *Gbrowse* [19] database and associated interface are also embedded in the platform.

### Data content and processing

The 12 pseudomolecules sequences representing the version 2.40 of the reference tomato genome, *S. lycopersicum* cv Heinz, together with the chromosome 0, which includes all the contig sequences not assigned to any other chromosome yet, and the corresponding gene annotation version 2.30 from the international Tomato Annotation Group (*iTAG*) [30], were downloaded from the *Solgenomics* website [36]. The genome sequences and the *iTAG* gene annotation were uploaded in the platform (Figure 1).

The *Illumina HiSeq 2000* [40] RNA-seq data collection of *Solanum lycopersicum* cv Heinz [30] was downloaded from the NCBI SRA archive [SRA: SRP010775]. This collection includes 20 libraries, each one representing one of two biological replicates from 10 different tissues and stages in physiological conditions.

The *Illumina HiSeq 2000* RNA-seq data collections from physiological conditions of *Solanum lycopersicum* cv Ailsa Craig [SRA: SRX098400], including 20 libraries [34], and of *Solanum pimpinellifolium* [SRA: SRP010775], including 8 libraries [30], were also downloaded from NCBI SRA archive [3].

### Illumina reads processing

Raw *Illumina* reads were cleaned from adaptor sequences and those with a quality lower than Q20 were discarded using *trim galore* [41]. Reads shorter than 20 bp were also discarded. Filtered and cleaned reads were indexed by *bowtie2* [9] and mapped onto the Tomato pseudomolecules using *Tophat2* [10], with default parameters (up to 2 mismatches and intron length of 50 to 500,000 nt). Ambiguous matches were filtered out, i.e. reads with multiple matches on the genome were eliminated.

The results of reads to genome mapping were then loaded into the *Gbrowse* system embedded in the platform.

### Gene expression analysis

The gene expression levels for the 34,727 tomato predicted mRNA loci, were defined by the *htseq-count* software [42], considering reads per each exon. Raw data counting and several normalization methods were considered. The normalized number of reads by the size factor normalization of each tissue/stage in comparison with the size factor of other tissues/stages and the Median (med) normalization technique implemented in the *DESeq* package [11] were also performed; the Reads Per Kilobases Per Million (RPKM) [27] normalization was also independently applied. The expression level results calculated from all mentioned normalization methods together with raw reads counting were uploaded in the platform.

### GO term to gene association

Gene to GO Terms associations were defined by combining two major reference collections: i) the GO reference collection of *S. lycopersicum* downloaded from the *BioMart* database [43] (release June 2014), ii) the results of the *S. lycopersicum* mRNA sequences *Blast2Go* [26] versus the *NCBI* non-redundant database (nr). The two datasets were then combined removing duplicated terms. The GO annotations associated to the *iTAG* genes were uploaded into the devoted section of the platform (Figure 1).

## Utility

### User interface and database access

**Figure 2:**
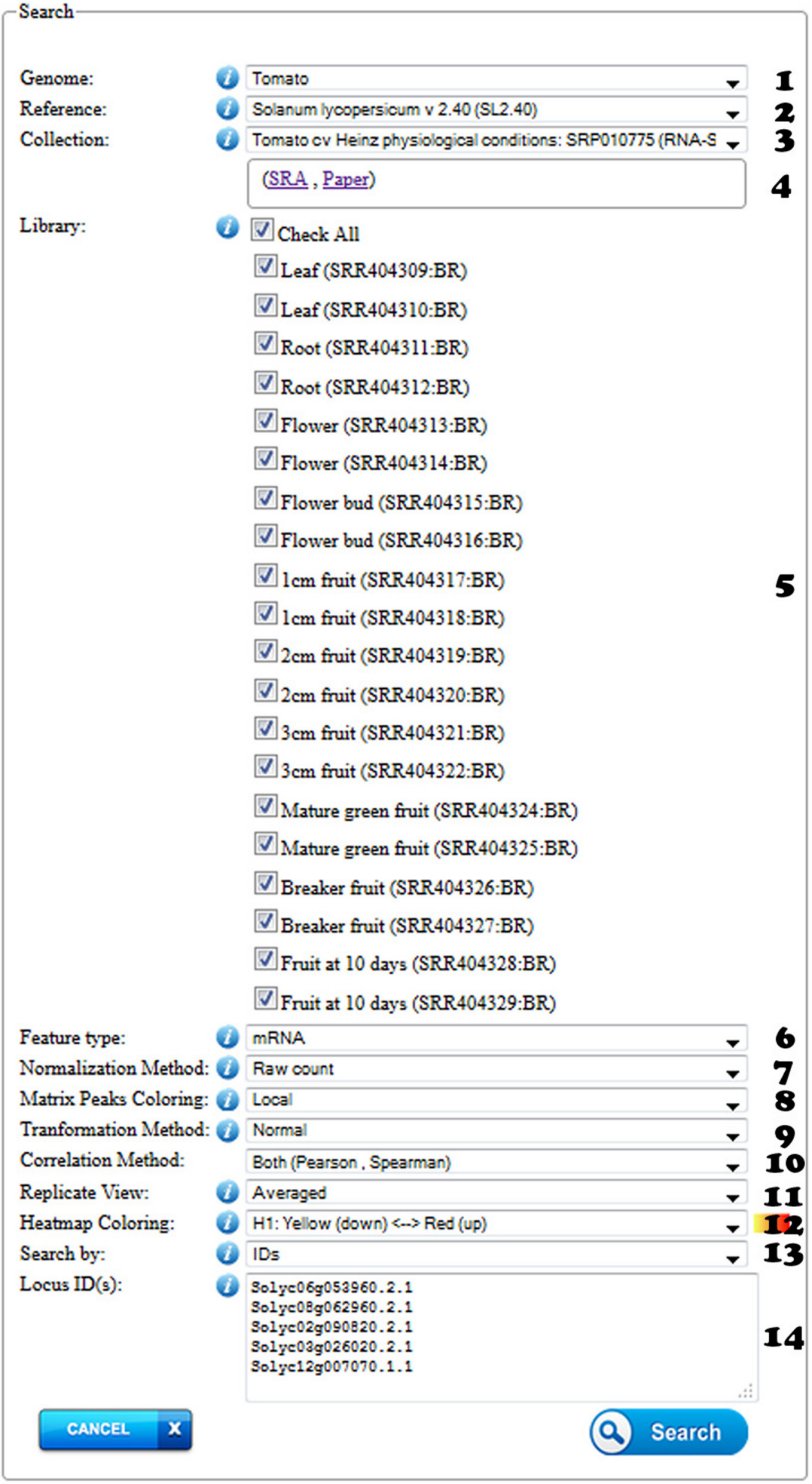
**Main sections provided in the** ***NexGenEx-*** **platform query form.** 1) Genome: the genome of interest, e.g. Tomato, 2) Reference: it indicates the reference genotype or cultivar sequences of interest, e.g. *Solanum lycopersicum* cv Heinz, version 2.40; 3) NGS collections available in the platform, e.g. the Heinz *Illumina* based RNA-seq collection in physiological condition; 4) link to the data source and paper for this collection; 5) available libraries (replicates/stages/tissues) included in the collection; 6) Feature type: represents the reference genome feature selected for read counting (e.g. mRNA, represents the exons in the locus); 7) Normalization method; 8) Matrix peak coloring, which defines the approach for color coding of the expression levels. This option assigns color frequencies to the cells of a heatmap view comparatively with the expression levels within the query result set (*local* *)* or within the whole selected libraries (*global* *)*. 9) Transformation method: expression levels or their log2 transformed results can be accessed; 10) Correlation method: Pearson product–moment correlation coefficient or Spareman’s correlation coefficient or Both; 11) Replicate view: defines the expression level by each libraries (*Separate*) or averaged between identical replicates (*Average*); 12) Heatmap coloring: different heatmap coloring combinations are provided for expression level visualization; 13) Search in: searchable fields can be one/multiple locus ids (IDs), or simple/multiple functional keywords with advanced selection options (Keyword), or genome regions (Region), and 14) the search area (Locus IDs/Functional keyword/Region): is the text area in which IDs or functional keywords may be listed, or a specific region of the genome may be specified. Accepted formats are described in the information pop-up from the website interface. “*Info”* buttons are available to support the users.

In Figure 2, we report the query page of the *NexGenEx-* platform. The figure shows the main sections users are provided with when consulting the platform content. In the *S. lycopersicum* cv Heinz dedicated partition (which is accessible selecting ***genome*** “Tomato”, ***reference*** “*S. lycopersicum* Version 2.40”) the three available gene expression collections (Heinz, Ailsa Craig, *S. pimpinellifolium*) can be selected in the ***collection*** field. Crosslinks to reference raw collections and to the papers presenting them are also provided.

### Results view

The *NexGenEx-Tom* platform enables users to investigate expression of the reference tomato genes in different tissues and developmental stages from different collections in physiological conditions. Users can exploit the platform to investigate on a specific gene, or a set of genes. The query can be based on a list of Gene Identifiers (IDs) in the form of *Solyc* identifiers (e.g. *Solyc01g00500*), or by indicating one or more functional keywords, or by specifying the boundaries of a chromosome region (indicating the specific directionality of transcription by selecting the *strand* option). Complex queries can be defined as indicated in the “***info***” links (Figure 2).

The web-based list of results is organized in an accordion view in which each result set can be investigated in its corresponding section/tab.

**Figure 3:**
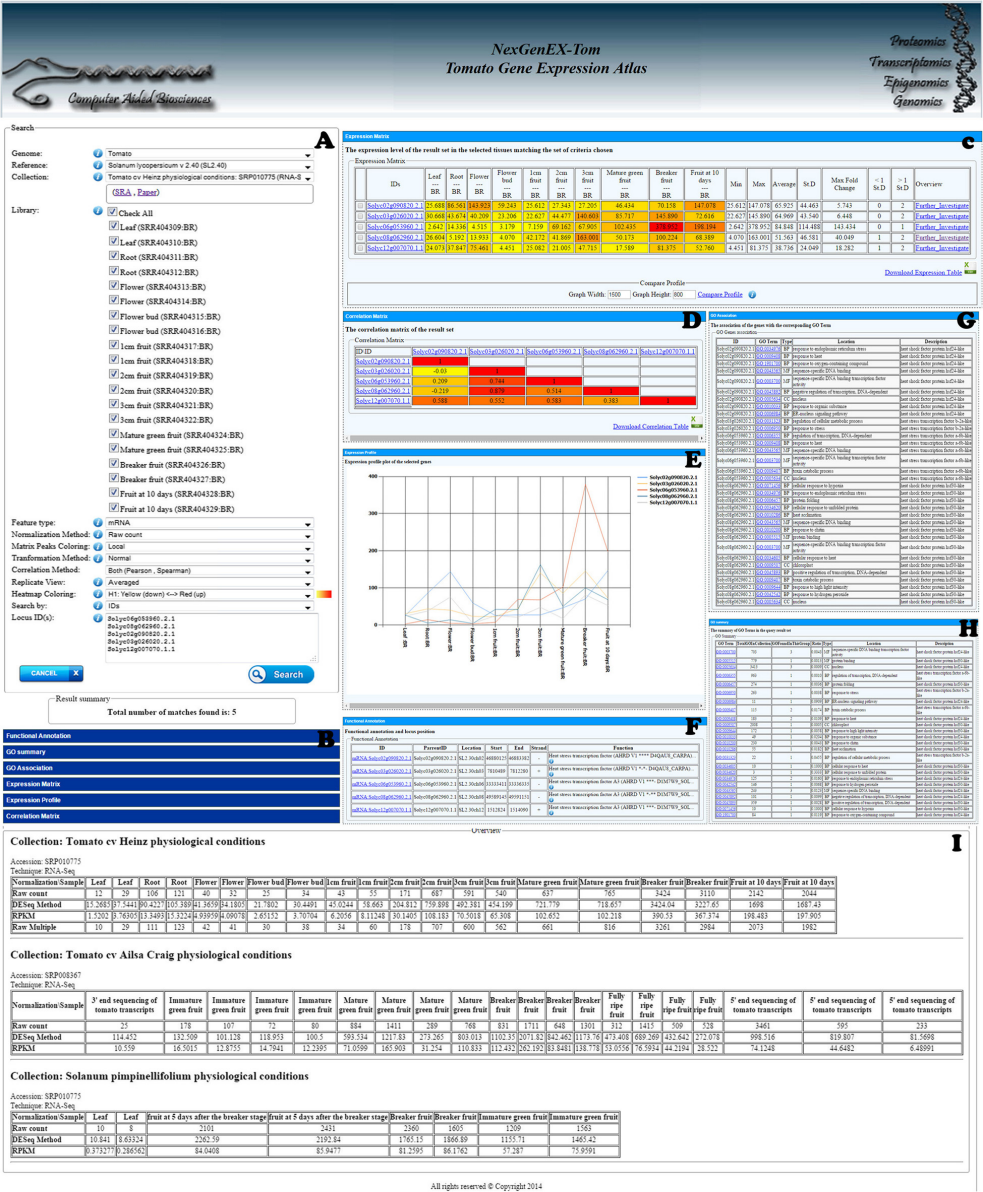
**Snapshots of results provided by the platform: A)** example query; **B)** accordion view of all results; **C)** expression matrix and profiling; **D)** correlation matrix; **E)** expression profiling plot; **F)** functional annotation and locus position on the genome; **G)** GO Terms summary table; **H)** GO Terms association table and **I)** Locus overview from all collections.

In Figure 3, an example query including a list of 5 gene IDs and the corresponding result views are shown.

### Annotation of the structure and functional annotation

By running the query in the system (Figure 3A), the list of the resulting loci associated to the query, including their functional annotation and accessory information from the current gene annotation is reported (Figure 3F).

### Expression matrix and profiling

The expression levels of each queried gene, based on the pre-settings of the query option provided by the user, can be investigated by the selected libraries, in the form of read counts per each locus, median normalized counts or RPKM. As an optional parameter, the average expression level of the replicates from each library can be investigated (***Replicate view*** set to ***Average***, in the query options). In the specific case of the Heinz library two replicates are included in the experiment (Figure 3C).

In addition, a complementary set of statistics, such as the minimum, maximum, average, standard deviation and maximum fold change of the expression levels of each locus in the selected libraries are provided. Moreover, the number of times each of the expression values exceeds the boundaries of one standard deviation from the average is also shown. This value permits to efficiently investigate locus specificities [44]. This section provides a general abstract of the loci expression level behavior in the selected libraries. Moreover, by selecting only 2 different tissues for specific gene sets, bi-comparison of the gene expression fold change are delivered permitting to identify the differential expression levels.

The expression matrix can also be downloaded in csv format

For each resulting gene, a button (***further investigate***) has been implemented which enables users to further investigate its gene expression in different conditions (Figure 3C). Indeed, by clicking on this link, the expression level of the corresponding gene will be reported for all the available NGS collections in the platform (Figure 3I), in all the available normalized forms, as calculated for each collection associated to the genome reference. This enables users to focus on the locus of interest with a complete overview of its behavior in any possible and available library per collection.

### Expression profiling plot

The Expression Profiling plot (Figure 3E) shows gene expression variability in different samples from the collection under investigation. This view depends on the number of libraries selected. The possibility to perform the analysis on specific collections of genes, selected by keyword or ID or by a genome region, allows the comparison of the expression profiles of several genes in a straightforward way.

### Heatmap visualization

Heatmaps provide a suitable view on gene expression levels. Customizable heatmaps are offered in the platform to highlight high- and low- expressed genes. This graphical approach is exploited in the platform to show the expression levels of one or multiple genes in different conditions. The data can be reported in the form of a matrix, where the level of expression can be marked by a specific color scaling, which may help to highlight high, medium and low levels of expression. The heatmap provided in the *NexGenEx-* platform (Figure 3C) can be defined by a ***local*** or a ***global*** scaling, according to the preferred selection in the Matrix Peak Coloring option (Figure 2.8). The “***local***” heatmap option provides the expression level coloring ranging from the lowest to the highest expression levels resulting from the query. This facilitates the comparison of the specific gene expression levels in the selected set. The “***global***” coloring option defines the coloring range on the basis of the lowest and highest expression levels in the whole libraries selected during the query. This enables users to identify the gene expression level when compared with the whole expression levels from all the genes in the selected library/ies.

### Correlation matrix

The Correlation matrix analysis illustrates the correlation between genes on the basis of the selected libraries (Figure 3D). The analysis can be based on the Pearson product–moment correlation coefficient, or on the Spearman's rank correlation coefficient, or both at the same time; and the resulting values fluctuates between −1 to 1, providing the negatively or positively correlated genes. The correlation matrix can be also downloaded in csv format.

### GO terms summary table and their association

As it is shown in Figure 3, a GO Term summary table and the gene to GO Term association to the queried genes is provided to the end users. Figure 3G shows an example of a resulting GO Term summary table of the list of occurring GO Terms, type of GO (in terms of CC: Cellular component, MF: Molecular Function and BP: Biological Process), GO location and the specific GO descriptions. In addition, the enrichment of the GO in the resulting gene set is sorted by p-value for further investigation purposes.

Moreover, the genes to GO Terms association table (Figure 3H) also provides the association list of the resulting genes with their GO Terms and their complete description. To further investigate the GO Terms, each GO is also linked to the *AmiGO* ontology and annotation database [45].

### Genome browser crosslink

**Figure 4:**
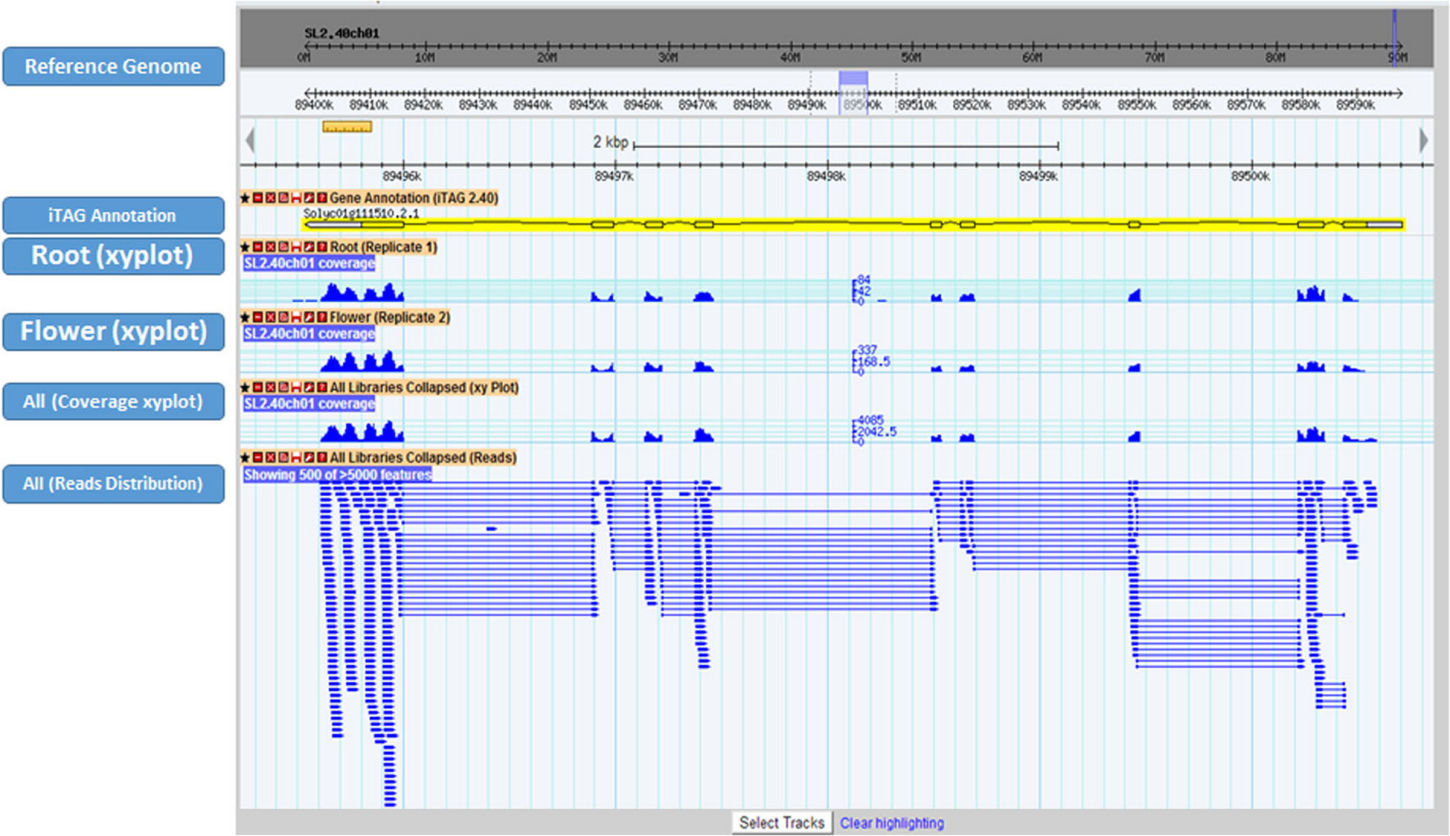
**Example of a** ***Gbrowse*** **based view.** The gene locus, the xyplot coverage of the NGS reads and their mapping along the selected locus are shown.

*NexGenEx-Tom* is enriched with an embedded, customized and updated genome browser interface [19]. The genome browser used, *Gbrowse*, permits a genome based investigation of the structure of the gene loci included in the database and can be accessed by the selection of each locus from the query set (Figure 4).

Expression profile of the reads mapped on the genome for each tissue/stages is provided in the form of read tracks and coverage plot (xyplot). Another track defined on the basis of all the reads from the available libraries (combined) is also added to provide a general overview of the locus expression for each collection.

Figure 4 shows an example of a *Gbrowse* based view offered by the platform. This view enables users with in-depth investigation of the selected loci and their associated pattern of expression in the form of reads distribution along the genome sequence. The *iTAG 2.30* gene annotation is also accessible through the *Gbrowse* partition. Specifically, the *NexGenEx-Tom Gbrowse* partition is also enriched with all the annotation tracks included in the *ISOL@* platform [46].

### A general overview on gene expression analysis from the Heinz genotype

An example of useful evidence the platform can provide is here reported, considering the Heinz genotype RNA-seq collection [30].

**Figure 5:**
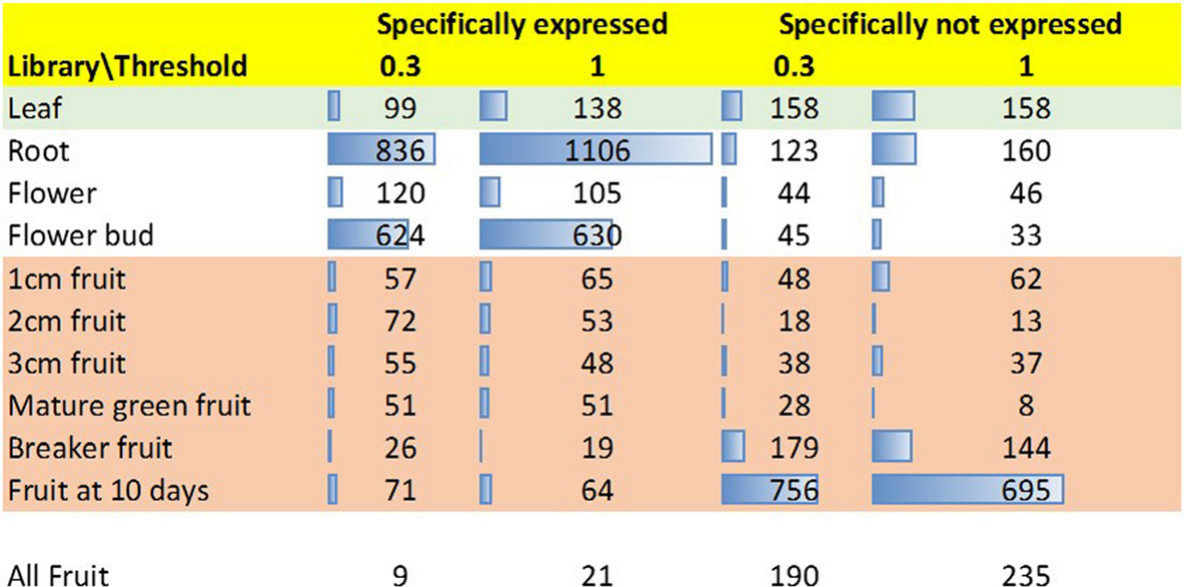
**Tissue specific genes in Heinz reference collection.** The number of genes with specific behavior is reported considering two different thresholds.

Our data analysis highlighted that among all 34727 *iTAG* annotated genes, 6412 genes show zero read mapped on the transcribed region of the gene locus when considering any of the libraries from each of the replicates of the presented Heinz atlas collection. Interestingly, the number of genes having no read mapped on their gene locus (zero expression level) in the paper of the tomato genome first release [30] is 5700. This is probably due to analytical approaches and highlights the importance of clear description of the methods used to reproduce the data. Moreover, 10025 genes show expression levels lower than 1 RPKM in all the tissues/stages, falling in the criteria to be defined as not expressed genes. Hence, in total, 24702 genes show expression level higher than 1 RPKM in at least one of the investigated physiological conditions. We also analyzed the number of genes that showed the expression level higher than specified thresholds (0.3 and 1 RPKM, respectively) only in one condition (defined as specifically expressed). On the other hand, we also reported the list of genes with expression level lower than the given thresholds only in a specific condition (defined as specifically not expressed). In Figure 5, we report the number of specifically expressed and specifically not expressed genes per conditions according to the different thresholds. The statistics shows that comparatively a large number of genes (1106) are specifically expressed in root while a significant number of genes (695) are tissue specifically not expressed in “fruit after 10 days”. This overview represents just a limited view of the power of this kind of platforms, including homo-specific and comparable collections.

## Discussion

Though gene expression of tomato tissues has been widely investigated in the years, thanks to both EST sequencing and different microarray platforms, the resulting collections are heterogeneous in terms of experimental approaches employed, genotypes investigated and conditions considered. This makes the reconciliation of the data hard and far from providing a gene expression atlas of the species. The recent release of NGS collections including data from several tissues and stages from physiological conditions for specific genotypes [30,34] contributed a relevant resource to be appropriately exploited for comparative analyses of gene expression patterns in tomato. This paves the way to solve key questions for this species, which is also considered a model for fruit ripening and development. Therefore, an exhaustive organization of the results from the processed collections in a web accessible computational environment, enriched with tools and methods for their explorations, still represents a need for the scientific community.

Many efforts have been undertaken to support the processing and the exploration of results from NGS based collections. Some approaches are mainly devoted to data visualization, as in the case of genome browser interfaces [22,18]. Other efforts result in platforms designed to face the complexity of bioinformatics analyses in genomics, offering free [13-15] or under payment tools [16,17].

The platforms may allow the setup of customizable workflows [13,14], or are based on pre-defined pipelines [15], providing services and methods for results investigation and reproducibility. However, though based on standards, the data processing requires, in most cases, user’s awareness on methods and parameters, as well as a constant updating on data requirements and novel tools, due to the fast evolution in the field.

While choosing the correct framework to tune the analyses and selecting the reliable resources to integrate in each specific context can be a challenge for the end-users, several platforms have been proposed to overcome these issues. Some efforts, therefore, such as *RNASeqExpressionBrowser* [20], mainly aimed to contribute to data integration and exploration thanks to suitable web interfaces for querying the RNA-seq expression results, that can be easily loaded as long as they are represented in a matrix-like format. In alternative, web based resources have been developed to offer immediate views on the results. For instance, in plants, several databases organizes NGS based collections [for the TIGR, soybean, Maize, TGRD, SGN and TFGD]. These platforms, each in a specific context, usually are devoted to organize the main NGS data for a species and, often, also analytical tools and appropriate query systems, possibly including other added-value resources in the specific genomics context.

Gene expression data from several technological approaches (ESTs, microarray, RNA-seq) dedicated to transcriptome analyses have been expanded during years of research in Tomato genomics. TFGD [35] offers a reference site to collect several of the tomato gene expression related collections, also including, per each RNA-seq based collection, tools and query interfaces to access gene lists and their RPKM normalized values per sample. Moreover, dedicated platforms to allow exploration of NGS reads mapped onto genome sequences, permitting the visualization of their distribution along each chromosome, are available at the *SGN* website [36], *ISOL@* [46], *TGDR* [37], *SPUD* [25].

However, suitably organized processed collections to offer enhanced and flexible NGS based analyses for tomato are still required. In this context, we implemented *NexGenEx-*. We aimed to offer processed collections, providing a user-friendly platform and tools for exploitation of gene expression analysis based on a genome reference and, possibly, on accessory information available from the species.

*NexGenEx-Tom* is the tomato dedicated partition of the *NexGenEx-* platform, specifically designed to allow investigations on NGS based transcriptome collections representative of different tissues/stages from different genotypes [30,34].

It offers a novel resource that permits to explore gene expression by library, by genotype, and comparatively between genotypes, highlighting possible peculiarities and specificities. This effort is also relevant as a reference at the light of the manifold similar experiments that are being undertaken in tomato.

The flexibility and the customizable bioinformatics supported by *NexGenEx-* enables different views and representations of the embedded results. The access to cleaned and processed data, differently normalized collections (such as the median normalized, not considering the length bias, and the RPKM, including the effect of the length bias), the rescaling based on the log2 transformation, the possibility to select one or multiple genes, from part or all libraries, considering or not the replicate contribution, provide a novel bioinformatics bench-work for immediate investigations and a user-friendly computational environment.

The statistical report section, including minimum, maximum, average, standard deviation and the maximum fold change of the expression levels in the selected libraries, also provides support to investigate specificities for the desired query set.

The query by Gene Identifier(s), or by Functional Keyword(s) or by specifying the boundaries of a chromosome region, based on an efficient data management system, permits complex conceptual browsing of the platform information content. Associated to the gene list and the corresponding functional annotation, an updated Gene Ontology report is provided.

A useful feature available in *NexGenEx-* is the possibility to straightforward investigate gene expression levels in different formats from all the libraries of several collections. This permits comparative analyses based on the same genome reference.

*NexGenEx-* also includes the direct link to a genome browser interface, thanks to the embedded *Gbrowse* interface [22]. This allows to visualize reads mapped to the genome by library, even when representing different replicates, integrated with the available genome annotation, offering a deep view on read to exon distribution, and, therefore, highlighting possible peculiarities such as alternative exons in a locus. Specifically, the *Gbrowse* database included in *NexGenEx-Tom* is also enriched with the data collected from the tomato genome browser implemented in *ISOL@* [46], permitting to enrich by several additional tracks the whole resource and further support genome based gene expression analyses.

Beyond providing a user-friendly interface to investigate comprehensive reference collections, the platform was designed with the aim to easily be expanded with other NGS based data. It can also integrate different genome releases, possibly from different cultivars or genotypes, but even from different species. As a consequence, though the platform is proposed as an effort in tomato genomics, it can represent a useful tool also for other similar species, permitting the gene based exploitation of these precious and challenging datasets even by bioinformatics non –expert users.

## Conclusions

The emergence of NGS technologies opened an unexpected precious window to a deeper understanding of genome organization and gene expression mechanisms, regulation and control. Their contribution is enhanced by suitable experimental project design, in the form of gene expression atlases that could highlight the coordination of the events that make the genome express its genetic information in physiological or alternative conditions. This strongly requires the corresponding organization of bioinformatics environments where the results from processed data are appropriately organized and may be straightforward investigated and compared even by users with limited experience in bioinformatics. *NexGenEx-* aims to give a contribution to this challenge and here we described its main peculiarities considering the partition *NexGenEx-Tom*, organizing tomato gene expression collections based on *Illumina* data.

*Solanum lycopersicum* cv Heinz has been selected to represent the reference tomato genome and also as a reference to support comparative analysis within the *Solanaceae* family. The availability of related consistent transcriptome collections, can further highlight the main functionality of this genome representing a profitable resource for all the interested scientific community.

In the presented version, *NexGenEx-Tom* aims to contribute to gene expression analyses in tomato offering three relevant processed NGS based transcriptome collections and user-friendly tools to permit their exploration.

Though our main purpose at this stage was to provide a bioinformatics environment for supporting genome wide investigations based on the reference tomato genome in physiological conditions, the platform architecture has been designed and is proposed as a model approach for similar efforts. Moreover, at the light of the rapid expansion of NGS based efforts, the platform is already organized to be expanded with other transcriptome collections, possibly from different genotypes, and eventually even from different species, representing a model strategy to face the challenge and the opportunity offered by these precious data resources.

## Availability and requirements

Access to *NexGenEx-Tom* is freely available for research purposes, for users from non-profit and academic organizations at http://www.cab.unina.it/NexGenEx-Tom.

The platform is designed in a role based architecture which also enables dedicated access to private data after authentication (password protected section) for collaborative efforts and to protect still unpublished results upon user specific requests. Interested users are invited to follow the contacts for agreements on the inclusions of their collections in the platforms as collaborative efforts or as a service.

The platform is also supported with specific Application Program Interfaces (APIs) to enable, upon request, interested developers to deploy in-house queries within their system, getting advantage of available data and analysis in the *NexGenEx-* system.
